# Growth Performance Can Be Increased Under High Nitrate and High Salt Stress Through Enhanced Nitrate Reductase Activity in *Arabidopsis* Anthocyanin Over-Producing Mutant Plants

**DOI:** 10.3389/fpls.2021.644455

**Published:** 2021-07-01

**Authors:** Ye Ji Lee, Won Je Lee, Quang Tri Le, Suk-Whan Hong, Hojoung Lee

**Affiliations:** ^1^Department of Plant Biotechnology, College of Life Sciences and Biotechnology, Korea University, Seoul, South Korea; ^2^Institute of Life Science and Natural Resources, Korea University, Seoul, South Korea; ^3^Department of Molecular Biotechnology, Bioenergy Research Center, College of Agriculture and Life Sciences, Chonnam National University, Gwangju, South Korea

**Keywords:** *Arabidopsis*, anthocyanin, nitrate, nitrate reductase, salt stress tolerance

## Abstract

Nitrogen is one of the most important macro-nutrients for plant growth and crop productivity. The amount of synthetic nitrogen fertilizers supplied to crops has dramatically increased, leading to a notable rise in crop yields. However, excessive nitrogen use has an enormous negative impact on ecosystems and human health through the emission of intense greenhouse gases, such as nitric oxide derived from the nitrate (NO_3_^–^) assimilation cascade. Additionally, owing to the development of extensive irrigation in agriculture, crops are known to suffer from high salt stress. The effect of excessive nitrogen fertilizer application has been studied in some crops, but the effect of high nitrate level and salt stress on plant stress tolerance has not been studied in detail. Therefore, in this study we aimed to study the effects of high concentrations of NO_3_^–^ on salt stress tolerance in *Arabidopsis*. In addition, since anthocyanin functions as a reactive oxygen species (ROS) scavenger under abiotic stress conditions, we investigated whether enhanced anthocyanin content helps *Arabidopsis* to withstand higher salt stress levels under high NO_3_^–^ concentrations by using *pap1-D/fls1ko* double mutant plants, which accumulate excessive amount of anthocyanin. We found that Col-0 plants are more sensitive to salt stress under high NO_3_^–^ concentrations. Although both the *pap1-D/fls1ko* and *fls1ko* plants accumulated higher anthocyanin levels and radical scavenging activities than Col-0 plants under both normal and salt stress conditions, the *fls1ko* plants exhibited much better growth than the *pap1-D/fls1ko* plants. It appears that the enhanced NR activities and transcript levels of *NIA1* and *NIA2* in *pap1-D/fls1ko* and *fls1ko* plants led to an increase in the synthesis of proteins and proline, which increases osmolytes against salt stress. Our results demonstrate that optimal levels of anthocyanin accumulation can enhance growth performance of plants under high NO_3_^–^ and salt stress conditions.

## Introduction

Recently, the demand for high quality vegetable crops has steadily increased due to global population growth and the rapid pace of economic development. Intensive greenhouse cultivation of vegetable crops is becoming common; greenhouse soil for vegetable cultivation typically has a high NO_3_^–^ content in agricultural systems (Shen, 2010). In greenhouse cultivation, constant single crop cultivation and, excessive nitrogen fertilizer application change the transformation process of soil nitrogen and accelerate the accumulation of NO_3_^–^ (Shen, 2010; Li et al., 2015). This not only causes serious environmental pollution (Schlesinger, 2009), but may also impact the stress tolerance and productivity of the plant itself. For example, the use of excessive nitrogen fertilizer was reported to negatively affect the fruit yield and quality in tomatoes and reduce agricultural sustainability (Ronga et al., 2020). Moreover, it has been reported that the use of excess N fertilizers in green houses in China each year has resulted in significant changes in soil chemical properties, including lower nitrogen use efficiency (Zhu et al., 2005; He et al., 2007), and soil secondary salinization and acidification (Shen et al., 2008; Shi et al., 2009). Nitrogen fertilizers that are overspread in arable land are known to leach into the hydrosphere, except for a certain amount absorbed by plants. However, the authors who investigated the fate of isotopically labeled nitrogen fertilizers in a three-decade-long in situ tracer experiment demonstrated that 61–65% of the applied fertilizer N is consumed by plants, while at least 12–15% remain in soil organic matter (Sebilo et al., 2013). Therefore, if nitrogen fertilizer is sprayed every year, nitrogen will continue to accumulate in the soil environment and affect the growth of plants. Moreover, studies have shown that in some vegetable growing areas in northern China, a single season N fertilizer input is over 300 kg N ha^–1^; this amount is almost twice as much as it is required for most plant crops (Zhang, 2005), with the NUE being only 33% (Song et al., 2009). The excess of N fertilizer applied to the fields results in the accumulation of a significant amount of nitrate within the soil profile (Shi et al., 2009). In addition, due to the development of extensive irrigation in agriculture, crops are known to suffer from high salt stress. When high nitrogen and salt stress conditions are present together, the growth of plants may be further atrophied, requiring specific agricultural protocols to optimally manage specific crop cultivation systems under these conditions. As the development and use of smart farm systems in plant factory technology progress, it is necessary to study the optimization of nitrogen fertilizer application required for the production of crops suitable for a specific purpose.

Anthocyanin, an important secondary metabolite in plants, helps in stress tolerance by functioning as a potent antioxidant molecule (Oh et al., 2011; Li et al., 2017; Yong et al., 2019). For example, when *UDP-glycosyltransferases* (*UGT79B2/B3*) were overexpressed, anthocyanins accumulated and increased the antioxidant activity, enhancing the ability of the plant to cope with abiotic stress (Xu and Rothstein, 2018), whereas *ugt79b2/b3* double mutant *Arabidopsis* plants exhibited reduced stress *tolerance* (Li et al., 2017). Anthocyanins are also known to help improve our health and are sold as health promotion supplements. Increasing the nitrogen concentration decreases the anthocyanin contents in plants, while decreasing the nitrogen concentration dramatically increases the anthocyanin contents (Scheible et al., 2004; Diaz et al., 2006). This suggests that nitrogen and anthocyanin concentrations are closely correlated in plants; however, the reason for this correlation and the underlying regulatory molecular mechanisms remain unclear. The MYB-bHLH-WD40 (MBW) complex plays an important role in the regulation of anthocyanin biosynthesis in plants (Petroni and Tonelli, 2011; Albert et al., 2014). The role of one of these components, the *MYB* gene, *AtPAP1* (*AtMYB75*), was demonstrated by the discovery of the *PRODUCTION OF ANTHOCYANIN PIGMENT 1-dominant* (*pap1-D*) mutant plants (Borevitz et al., 2000). The *pap1-D* mutations obtained through activation tagging are known to cause overexpression of *PAP1* and over-accumulation of anthocyanins (Borevitz et al., 2000). Previously, we had generated a double mutant plant that lacks the *FLS1* gene (*fls1ko* mutant) in the *pap1-D* background (*pap1-D/fls1ko* plants) to further increase the anthocyanin content (Lee et al., 2016), because flavonol synthase (FLS) converts dihydrokaempferol into flavonols which are otherwise converted into anthocyanins via Dihydroflavonol-4-reductase (DFR).

As described above, the use of excess nitrogen fertilizers has been studied in several crops (Ronga et al., 2020), but many of the effects on plants remain elusive. Therefore, we investigated how the stress tolerance of *Arabidopsis* changes when high concentrations of nitrate are treated together with salt stress. We also questioned whether enhanced anthocyanin content could help *Arabidopsis* to further withstand salt stress in the presence of high NO_3_^–^ concentrations. In this study, we found that Col-0 plants were more sensitive to salt stress under high NO_3_^–^ conditions. High NO_3_^–^ conditions resulted in inhibition of the synthesis of anthocyanin, which act as excellent antioxidants in the abiotic stress responses of plants. We observed that although both the *pap1-D/fls1ko* and *fls1ko* plants accumulated higher anthocyanin levels than Col-0 plants under both normal and salt stress conditions, the *fls1ko* plants exhibited much better growth than the *pap1-D/fls1ko* plants. Here, we report the possible mechanism of the enhanced growth performance of *pap1-D/fls1ko* seedlings in response to salt stress under high NO_3_^–^ conditions.

## Materials and Methods

### Plants Culture Conditions and Generation of the *pap1- D/fls1ko* Plants

*Arabidopsis thaliana* wild-type, Columbia (Col-0) were used as control plants. The T-DNA inserted *ttg1ko* and the, EMS mutant, *fls1ko* were obtained from the *Arabidopsis* Information Resource (TAIR)^1^. To construct *pap1-D/fls1ko* plants, *pap1-D* and *fls1ko* plants were crossed producing homozygous F_3_ plants (Lee et al., 2016). The seeds were sterilized and stored for 3 days at 4°C. The seeds were germinated and grown on 10 mM NO_3_^–^ supplied with nitrogen-free half-strength Murashige and Skoog (MS) medium. Each plant was raised in a growth chamber under the following conditions: 16 h light/8 h dark cycle, 23°C, 50–55 μmol photons m^–2^s^–1^, and 70% humidity. The prepared 10 mM NO_3_^–^ half-strength MS medium contained 2% sucrose and 0.5% Phytagel. The pH of the medium was adjusted to 5.8. The seedlings were treated in the nitrogen-free half-strength MS medium with various concentrations of sodium chloride (NaCl) and potassium nitrate (KNO_3_).

### Measurement of Chlorophyll Contents

The chlorophyll contents were detected by spectrophotometry. The chlorophyll contents were extracted from 9-day-old seedlings. First, 50 mg of each samples were treated with various concentrations of NaCl and KNO_3_. Then, the plants were ground into a fine powder in liquid nitrogen. The prepared samples were treated with 700 μL of an 80% acetone solution at 21°C in a 1.5 mL tube. The solutions were mixed in the dark for 30 min to prevent chlorophyll damage. The mixture was centrifuged at 3,000 rpm at 4°C for 15 min. The absorbance was measured at 663 and 645 nm. Statistical analyses were performed by factorial ANOVA, followed by Tukey’s test for comparisons of means at least at the 95% confidence level. The following equations were used to estimate the chlorophyll concentrations:

$$\begin{array}{c} \mathrm{Chlorophylla}\,\left(\mathrm{mg}/g\right)=\left[12.7\times \left(\mathrm{A663}\right)-2.69\times \left(\mathrm{A645}\right)\right]\\ \times V/1,000\times W \end{array}$$

$$\begin{array}{c} \mathrm{Chlorophyllb}\,\left(\mathrm{mg}/g\right)=\left[22.9\times \left(\mathrm{A645}\right)-4.86\times \left(\mathrm{A663}\right)\right]\\ \times V/1,000\times W \end{array}$$

$$\begin{array}{c} \mathrm{Totalchlorophyll}\left(\mathrm{mg}/g\right)=[8.02\times \left(\mathrm{A663}\right)+20.20\\ \times \left(A645\right)]\times V/1,000\times W \end{array}$$

where (V is the volume of the extract, and W is the: weight of fresh leaves).

### Measurement of Anthocyanin Contents

Four-day-old seedlings were used for the anthocyanin analysis after treatment with 10, 25, and 50 mM KNO_3_ and 175 mM NaCl for 24 h. Here, 300 L of 80% methanol and 5% HCl was added to 4-day-old ground plant tissue and the anthocyanin was extracted overnight in a dark refrigerator. After this, 200 L distilled water (DW) and 500 L of chloroform were added to each tube. After centrifugation at 13,000 rpm for 20 min, the extracts were moved to new tubes and the amount of anthocyanin was quantified photometrically (DU 640 spectrophotometer; Beckman Instruments Beijing, China). The seedlings were collected for determination of the anthocyanin content at 535 and 650 nm using a spectrophotometer or plate reader. The blank sample comprised 480 L methanol 1% HCl and 320 L DW in a total volume of 800 L. All statistical analyses were performed using factorial ANOVA, followed by Tukey’s test for comparison of the means at the 95% confidence level.

### Quantitative Reverse Transcription Polymerase Chain Reaction

Each of seedlings was grown in the control medium for 9 days and then transferred to each concentration of NaCl and KNO_3_ with nitrogen-free medium for 6 h. Then the total RNA was isolated form these samples by using TRIzol (total RNA isolation reagent, Thermo Fisher Scientific) and checked the RNA quality by loading 3 μL of the extracted RNA sample to RNA loading gel. For the first strand, the cDNA was synthesized as described by Lee et al. (2016) by using MMLV reverse transcriptase. The strand-specific cDNA was used for quantitative reverse transcription polymerase chain reaction (qRT-PCR) analysis using a primer. qRT-PCR was performed by using EvaGreen 2 × qPCR MasterMix (Applied Biological Materials, Inc., Richmond, Canada) and Bio-Rad CFX manager program. AtActin2 and UBQ10 were used as the internal controls because they are known housekeeping genes. The primer sequences are listed in Supplementary Table 1. All statistical analyses were performed using one-way ANOVA, followed by Tukey’s test for comparison of means at the 95% confidence level.

### Measurement of Proline Contents

The proline content was measured as described previously (Bates et al., 1973). Briefly, proline was isolated from 100 mg of plants by grinding in 1 ml of 3% sulfosalicylic acid. A 200 μl aliquot of this extract was then reacted with a mixture of 100 μl of the ninhydrin reagent (80% glacial acetic acid, 6.8% phosphoric acid, and 70.17 mM ninhydrin) for 60 min at 100°C. Immersion in an ice bath was used to end the reaction; the reaction mixture was treated with 200 μl of toluene and vortexed. The absorbance rate of the toluene layer was measured at 520 nm in a UV/VIS spectrophotometer. The Proline concentration was extrapolated from a standard curve, and calculated on an FW basis as follows: [(ng proline ml^–1^ × ml extraction buffer)/115.5 ng nmol] g^–1^ sample = nmol proline g^–1^ FW material.

### Measurement of Sucrose Contents

Each 9-day-old seedling was supplemented with various concentrations of NaCl with KNO_3_ for 24 h. Then, 100 mg of ground tissue was used for sucrose extraction by adding 1 mL of 80% HEPES buffered ethanol for 10 min. The pH was adjusted to 7.8. Using glucose and sucrose assay kits (Sigma-Aldrich, #SCA20), 100 mg of the prepared samples were analyzed, according to the manufacturer’s protocols. The extracted solutions were then filtered by through a 20-nm nylon mesh. Then the extractions were centrifuged at 4,000 g for 5 min. The sucrose contents were then expressed as the sucrose concentration in the fresh weight (μg/mg) and the number of seedlings. The statistical analyses were performed using a factorial ANOVA, followed by Tukey’s test for comparison of means at the 95% confidence level.

### Measurement of NO_3_^–^ Contents

Each of the seedlings were supplemented with various concentration of NaCl and KNO_3_ for 1 day. The 50 mg samples of whole 9-day-old seedlings were used to isolate the NO_3_^–^ contents. The prepared samples were washed with distilled water (DW) and ground with liquid nitrogen. DW was added to the samples for 1 mL, and then adding the boiling distilled water for 20 min. The sample was centrifuged at 13,000 rpm at 4°C for 10 min. The tube was vortexed after 100 μL of the supernatant was treated with 400 μL of salicylic sulfate acid in a 15-mL Falcon tube. The samples were incubated at RT for 30 min. The samples were mixed with 9.5 mL of 8% NaOH solution and cooled down at 4°C for 5 min. The NO_3_^–^ levels were calculated from the absorbance at 410 nm. The statistical analyses were performed using a factorial ANOVA, followed by Tukey’s test for comparison of the means at the 95% confidence level.

### Nitrate Reductase Assay

Seedlings of 9-day-old Col-0, *pap1-D/fls1ko*, *fls1ko*, and *ttg1ko* plants were exposed to 10, 25, and 50 mM KNO_3_ with 175 mM NaCl for 1 d. Whole seedlings were used for the nitrate reductase assay. Nitrate reductase was extracted and calculated by using the NR Assay Kit (BC0080, SolarBio, Beijing, China). Each 50 mg seedling was supplied with 1 mL of the extraction solution and centrifuged at 4,000 rpm for 10 min. The supernatant was collected and analyzed further. The absorbance at 520 nm was used to calculate the nitrate reductase activity. The statistical analyses were performed using factorial ANOVA, followed by Tukey’s test for comparison of means at the 95% confidence level.

### BCA Assay for Protein Level Determination

Each seedling was exposed to various concentrations of NaCl and KNO_3_ at 9 days after growth under control conditions, and then proteins were extracted. PRO-PREP^TM^ Protein Extraction Solution (500 μL) was added to the samples after they were ground, and then they were vortexed. The samples were then incubated at −20°C for 20–30 min for cell-lysis, after which they were centrifuged for 5 min at 13,000 rpm and at, 4°C. The supernatant was transferred to a new e-tube, and was stored at −20°C for use in further experiments. For protein analysis, BCA (Merck millipore, United States) reagent was used. The prepared samples were heated for 30 min at 37°C. Then 25 μL of each standard or sample replicate was pipetted into a microplate well. After this 200 μL of the working reagent was added to each well and mixed for 30 s, and the plate was incubated at 37°C for 30 min for the standard assay (while the enhanced assay required 30 min incubations at 60°C). The samples were cooled at room temperature (10 min for the standard assay, 15 min for enhanced assay), and the absorbance at 562 nm was promptly determined, using a plate reader. Statistical analyses were performed using a factorial ANOVA, followed by a Tukey’s test for comparison of the means at the 95% confidence level.

### DAB Staining and DPPH Radical Scavenging Assay

3,3’-Diaminobenzidine (DAB) (D5637, Sigma-Aldrich), the H_2_O_2_ staining agent, was dissolved in DW and adjusted to pH 3.8 with KOH. The DAB solution was freshly prepared in order to avoid auto-oxidation. Three 2-week-old seedlings were transferred to 10, 25, and 50 mM KNO_3_ with 175 mM NaCl for 24 h. Prior to the transfer, seedlings were immersed and infiltrated under vacuum with 1.25 mg/ml DAB staining solution. Stained plants were bleached in acetic acid-glycerol-ethanol solution at 100°C for 5 min, and then stored in glycerol-ethanol solution. H_2_O_2_ was visualized as brown color due to DAB polymerization.

For DPPH radical scavenging assay, free radical scavenging ability of the plant extracts was tested by DPPH radical scavenging assay as described by Desmarchelier et al. (1997). A solution of 0.1 mM DPPH in methanol was prepared, and 2.4 mL of this solution was mixed with 1.6 mL of extract in methanol at different concentrations (12.5–150 μg/mL). After votrexing the reaction mixture, it was left in the dark at RT for 30 min. The absorbance of the mixture was measured spectrophotometrically at 517 nm. Percentage DPPH radical scavenging activity was calculated by the following equation: where A0 is the absorbance of the control, and A1 is the absorbance of the extracts/standard. Then % of inhibition was plotted against concentration, and from the graph IC50 was calculated. The experiment was repeated three times at each concentration. Statistical analyses were performed using a factorial ANOVA, followed by a Tukey’s test for comparison of the means at the 95% confidence level.

### Measurement of Sodium and Potassium Contents

Ten-day-old seedlings were treated with 10, 25, and 50 mM KNO_3_ with 175 mM NaCl for 24 h. Whole seedlings were used for the Na^+^ and K^+^ measurement. Then the 100 mg of samples were rinsed with deionized water and then each sample was dissolved in 0.6 ml of nitric acid in glass test tubes at 120°C for 2 h. After this, 0.4 ml of 60% (v/v) HClO_4_ was added to each glass tube. The samples were incubated at 150°C for 2 h until total sample volume is reduced to ≤0.5 ml. The samples were cooled down at room temperature and deionized water was added to samples up to 5 ml. Perkin-Elmer Optima 2000 DV inductively coupled plasma optical emission spectrometer (ICP-OES) was used to measure the concentration of sodium and potassium according to the manufacturer’s instruction. The statistical analyses were performed using a factorial ANOVA, followed by Tukey’s test for comparison of means at the 95% confidence level.

### Gene Accession Numbers

The gene sequences reported in this article can be found in the *Arabidopsis* Information Resource^2^ under the following accession numbers: *LBD37* (AT5G67420), *LBD38* (AT3G49940), *LBD39* (AT4G37540), *NRT1.1* (AT1G12110), *NRT 2.1* (AT1G08090), *NIA1* (AT1G77760), *NIA2* (AT1G37130), *CHI* (AT2G43570), *PAP1* (AT1G56650), and *MYBL2* (AT1G71030), *RD29A* (AT5G52310), *KIN2* (AT2G02800), *RD22* (AT5G25610), *COR15B* (AT2G42530), *DREB2A* (AT5G05410).

## Results

### Anthocyanin Over-Accumulation Helps to Resist High Salt Stress Under High NO_3_^–^ Conditions in *Arabidopsis*

**FIGURE 1:**
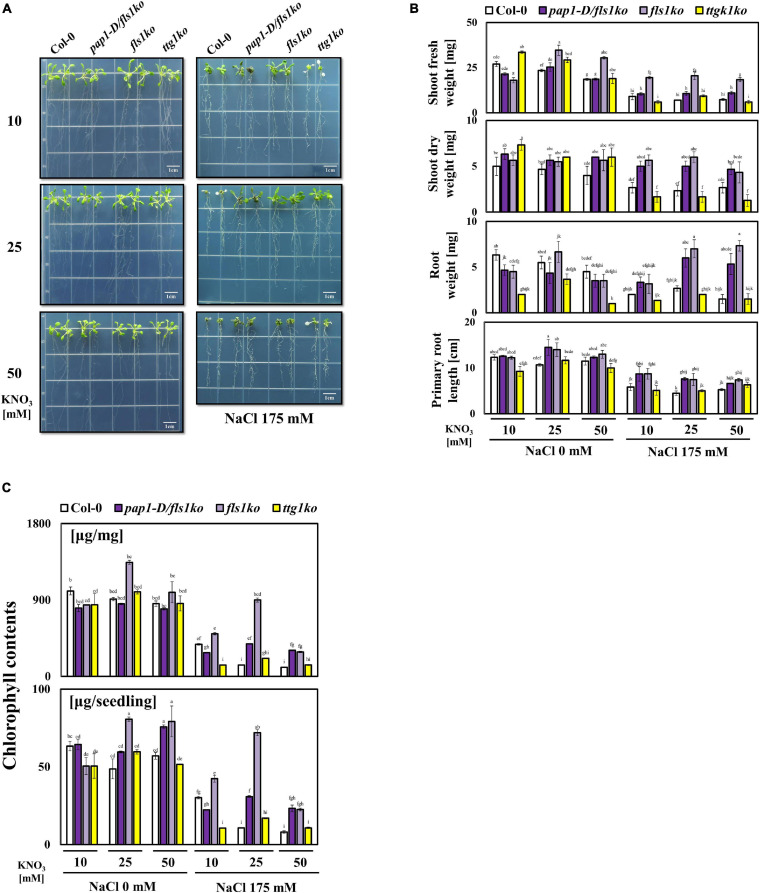
Growth phenotype of anthocyanin related mutants in response to various NO_3_^–^ combinations under salt stress conditions. **(A)** After their growth in normal 10 mM NO_3_^–^ medium, 9-day-old Col-0, *pap1-D/fls1ko*, *fls1ko*, and *ttg1ko* plants were transferred to 10, 25, and 50 mM KNO_3_ treated media as the control and to media with the same NO_3_^–^ concentrations along with 175 mM NaCl. The seedlings were grown in normal medium for 9 days, then moved to test medium for another 12 days; the seedlings were moved back to normal medium and grown for 5 more days to recover before taking pictures. **(B)** The shoot fresh weight, shoot dry weight, root weight, and primary root length of each seedlings were calculated to quantify the growth phenotype of the plants mentioned in **(A)**. The weight measurement of seedlings was carried out by using the plants harvested after growing 9-day-old plants in each medium for 12 days, then transferring to the 10 mM KNO_3_ medium for recovery for 5 days. **(C)** The chlorophyll contents of 9-day-old Col-0, *pap1-D/fls1ko*, *fls1ko*, and *ttg1ko* plants were measured after treatment with 10, 25, and 50 mM KNO_3_ and 175 mM NaCl for 24 h and are shown per fresh weight (mg). Three independent experiments were conducted, and the data were subjected to a factorial ANOVA, followed by Tukey’s test (*P* < 0.05). The letters above the columns indicate significant differences. Bars represent the standard errors.

To observe how the growth of plants changed and to investigate the effect of anthocyanin when salt stress and high nitrate conditions co-occurred, we examined the growth performance of Col-0, *pap1-D/fls1ko*, *fls1ko*, and *ttg1ko* plants in 175 mM NaCl combined with various NO_3_^–^ concentrations. The 4-day-old seedlings of each type from the control medium (10 mM NO_3_^–^, half-strength MS) were transferred to a medium with salt and high NO_3_^–^ content (KNO_3_ 10, 25, and 50 mM). All plants grew slightly smaller at 50 mM than at 10 mM NO_3_^–^ condition (Figure 1A). We found that the shoot fresh and dry weight and root weights of Col-0 decreased significantly with high level of nitrate (Figure 1B). However, in the case of *pap1-D/fls1ko, fls1ko* and *ttg1ko* plants, the growth phenotype was different from Col-0. In particular, in the case of *fls1ko*, the shoot weight was significantly higher than that of Col-0 at 25 and 50 mM than at 10 mM nitrate condition. Next, to reveal the effect of high nitrate on the salt stress tolerance of plants, the growth of Col-0, *pap1-D/fls1ko, fls1ko*, and *ttg1ko* plants was examined in medium with 10, 25, or 50 mM nitrate along with 175 mM NaCl; Col-0 and the *ttg1ko* plants showed a reduced growth performance and particularly suffered from a high NO_3_^–^ content (50 mM) under salt stress conditions, whereas we found that the shoot dry weight, as well as the shoot and root weights and primary root length, increased in the *pap1-D/fls1ko* and *fls1ko* plants compared with those of Col-0 (Figure 1B). The *pap1-D/fls1ko* and *fls1ko* plants showed more purple leaves and longer primary root length than Col-0 and *ttg1ko* plants. The *ttg1ko* plants had less purple pigment in their leaves with a shorter root length than Col-0, *pap1-D/fls1ko*, and *fls1ko* plants. As shown in Figure 1C), although treatment with high NO_3_^–^ concentration and 175 mM NaCl resulted in decreased chlorophyll contents in all plant types we examined, *pap1-D/fls1ko* and *fls1ko* plants accumulated more chlorophyll contents compared with Col-0 and *ttg1ko* plants.

**FIGURE 2:**
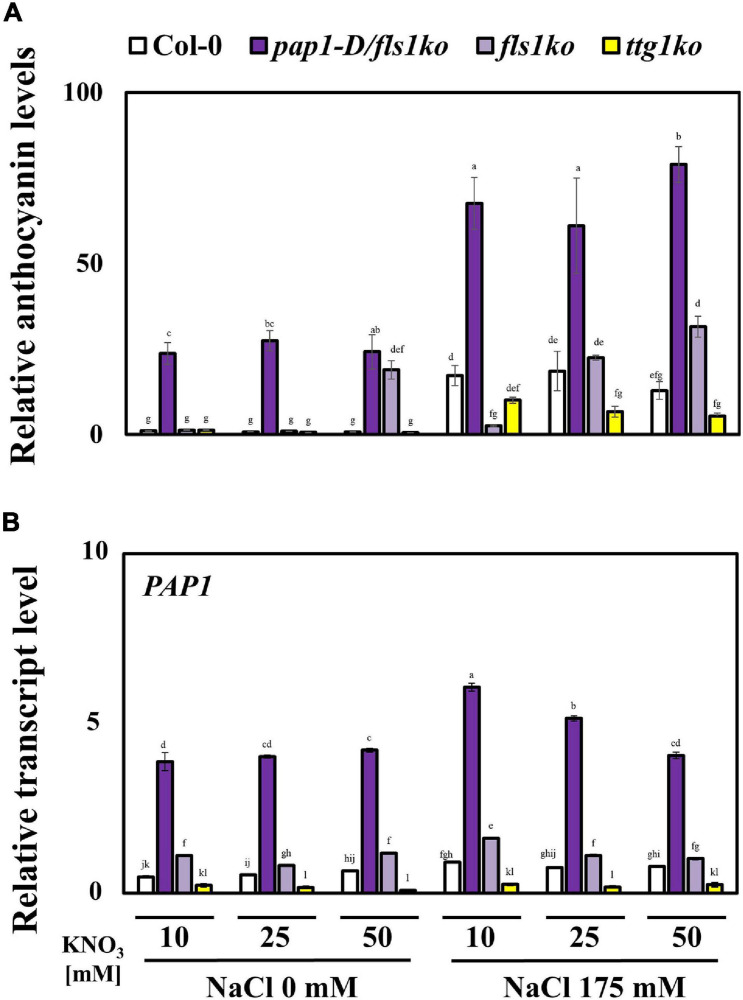
Anthocyanin contents and related gene transcript levels in plants after treatment with high concentrations of NO_3_^–^ under salt stress conditions. **(A)** The anthocyanin contents were measured in 4-day-old Col-0, *pap1-D/fls1ko*, *fls1ko*, and *ttg1ko* plants after they were treated with 10, 25, and 50 mM KNO_3_ and 175 mM NaCl for 24 h. The purple anthocyanin pigment was calculated by color-spectrometric absorbance measurement. **(B)** Nine-day-old Col-0, *pap1-D/fls1ko*, *fls1ko*, and *ttg1ko* plants were treated with 10, 25, and 50 mM KNO_3_ and 175 mM NaCl for 6 h and the RNA was extracted. The relative transcription levels of the anthocyanin biosynthesis gene, *PAP1* (anthocyanin-related transcription factor) were confirmed by using each cDNA sample. *Actin2* was used as an internal control. Three independent experiments were conducted, and the data were subjected to a factorial ANOVA, followed by Tukey’s test (*P* < 0.05). The letters above the columns indicate significant differences. Bars represent the standard errors.

PAP1 is a well-known transcription factor in the anthocyanin biosynthesis pathway in plants (Zuluaga et al., 2008). We investigated whether the accumulation of high levels of anthocyanin affected plant tolerance to high NO_3_^–^ and salt stress conditions. First, we determined the anthocyanin contents in 4-day-old Col-0, *pap1-D/fls1ko*, *fls1ko*, and *ttg1ko* plants after treatment with salt at various NO_3_^–^ concentrations for 24 h (Figure 2A). The anthocyanin levels decreased in response to the high NO_3_^–^ content; however, *pap1-D/fls1ko* and *fls1ko* plants accumulated higher levels of anthocyanin, whereas *ttg1ko* plants accumulated less than Col-0 plants. In order to investigate whether the difference in the potassium concentration affected plant growth according to different nitrogen concentrations, potassium chloride (KCl) was added to the nitrate 5 mM medium at concentrations of 5, 15, 25, and 45 mM. There was no significant difference in plant growth among these conditions (Supplementary Figure 1). In addition, the concentration of anthocyanin in plants treated with the KCl medium was also measured, and it was confirmed that the concentration of KCl did not cause significant difference (Supplementary Figure 2).

To investigate the reason for the reduced anthocyanin levels in response to high NO_3_^–^ concentrations even under salt stress conditions, we determined the transcript levels of anthocyanin biosynthesis inhibition-related genes after treatment of 9-day-old seedlings with 10, 25, and 50 mM NO_3_^–^ and 175 mM NaCl for 6 h (Supplementary Figure 3). Among the many known anthocyanin-related negative regulators in plants, the lateral organ boundary domain (*LBD*) genes, *LBD*37 and *LBD*39, showed up-regulation in Col-0 plants following treatment with high concentrations of NO_3_^–^, but the *pap1-D/fls1ko* and *fls1ko* plants also showed similar or higher transcript levels compared with the Col-0 plants (Supplementary Figure 3). The *MYBL2*, which acts as negative regulator of anthocyanin biosynthesis by inhibiting the MBW complex formation, was also up-regulated in Col-0 plants in response to the high NO_3_^–^ content; however, *pap1-D/fls1ko* plants showed significantly higher transcript levels compared with Col-0, *fls1ko*, and *ttg1ko* plants. We next examined the transcript levels of positive regulators of anthocyanin biosynthesis. which was the *PAP1*, a transcription factor of the anthocyanin biosynthesis enzyme genes (Figure 2B). The relative transcript level of *PAP1* in each of the seedlings was down-regulated in response to high NO_3_^–^ concentrations, a pattern similar to that of the anthocyanin contents in seedlings. These findings suggested that the reduced anthocyanin accumulation in response to high NO_3_^–^ concentrations under salt stress could be due to combined regulation of the enhanced transcription of negative regulators along with reduced transcription of the positive regulators of anthocyanin biosynthesis.

### Proline Contents Significantly Increased Under High Salinity and High NO_3_^–^ Conditions Compared With That Under Normal Conditions

**FIGURE 3:**
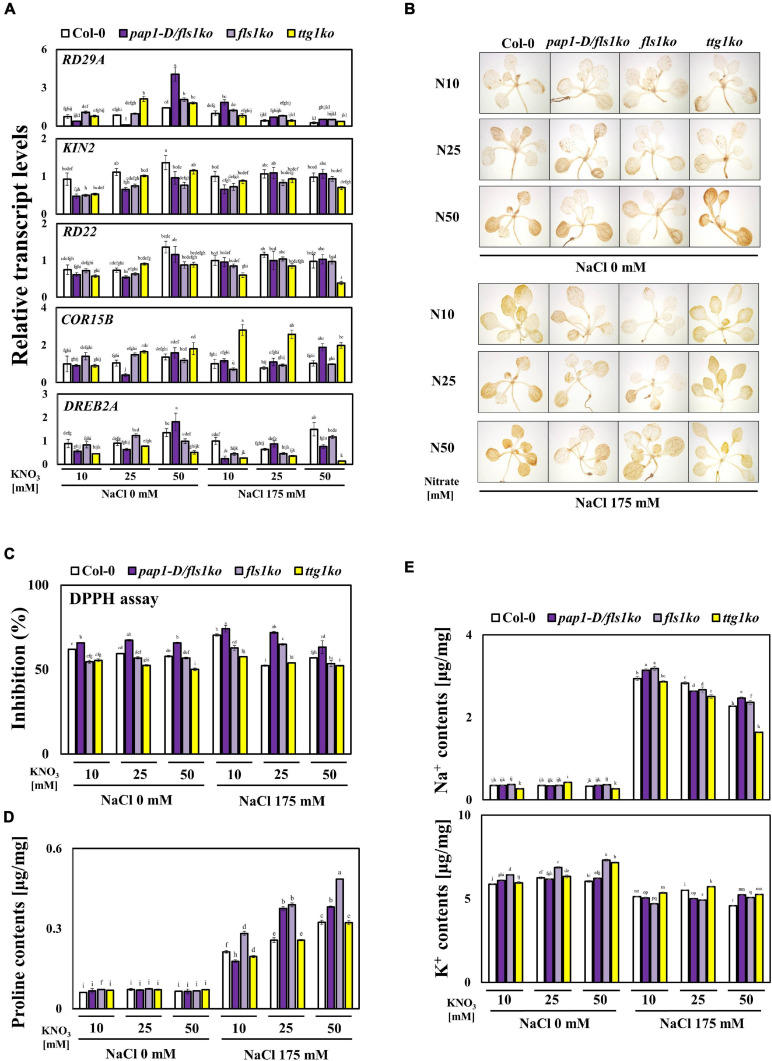
Altered ABA-related genes transcript levels, DAB staining, proline contents, and ion contents in *pap1-D/fls1ko* and *fls1ko* plants under high NO_3_^–^ and salt conditions. **(A)** Nine-day-old Col-0, *pap1-D/fls1ko*, *fls1ko*, and *ttg1ko* plants grown in normal media were treated with 10, 25, and 50 mM KNO_3_ and 175 mM NaCl for 6 h, and the RNA was extracted. The relative transcript levels of *RD29A*, *KIN2*, *RD22*, *COR15B*, and *DREB2A* were confirmed by using each cDNA sample. *Actin2* was used as an internal control. **(B)** Nine-day old seedlings were supplied to 10, 25, and 50 mM KNO_3_ and 175 mM NaCl for 24 h, and DAB staining was conducted. **(C)** Nine-day old seedlings were supplied to 10, 25, and 50 mM KNO_3_ and 175 mM NaCl for 24 h, and DPPH assay was conducted. **(D)** Nine-day-old seedlings were subjected to 10, 25, and 50 mM KNO_3_ and 175 mM NaCl for 24 h, and the proline content was calculated. **(E)** Nine-day old seedlings were supplied to 10, 25, and 50 mM KNO_3_ and 175 mM NaCl for 24 h then Na^+^ and K^+^ ion were measured from each seedling. Three independent experiments were conducted, and the data were subjected to a factorial ANOVA, followed by Tukey’s test (*P* < 0.05). The letters above the columns indicate significant differences. Bars represent the standard errors.

Since the *pap1-D/fls1ko* and *fls1ko* plants exhibited a better growth performance than Col-0 (Figure 1), we decided to examine the various salt stress responses of Col-0, *pap1-D/fls1ko*, *fls1ko*, and *ttg1ko* plants against salt stress compared to Col-0 in the presence of high nitrate level. First, we examined the transcript levels of the abiotic-stress related marker genes, *RD29A*, *KIN2*, *RD22*, *COR15B*, and *DREB2A* (Figure 3A). In Col-0 plants, the transcript levels of *KIN2*, *RD22*, *COR15B*, and *DREB2A* slightly increased following treatment with different concentrations of NO_3_^–^. However, we were not able to see significant difference in the transcript levels of these genes among Col-0, the *pap1-D/fls1ko* and *fls1ko* plants (Figure 3A). Under high salt and high nitrate conditions, the transcript levels of *COR15b* exhibited a significant increase in *ttg1ko* plants compared to Col-0, although the mechanism underlying this is currently unknown. Modulation of reactive oxygen species (ROS) scavenging systems can lead to enhanced tolerance to oxidative stresses imposed by many biotic and abiotic stresses (Pastori and Foyer, 2002). Thus, we examined the H_2_O_2_ level in the Col-0, the *pap1-D/fls1ko, fls1ko* and *ttg1ko* plants (Figure 3B). With DAB staining, the intensities of the stain appears slightly lower in the *pap1-D/fls1ko and fls1ko* than Col-0 in high nitrate and high salt conditions (Figure 3B). When we quantitatively measured anti-oxidant levels using DPPH assay, the radical scavenging activities were higher in the *pap1-D/fls1ko* and *fls1ko* plants than in Col-0 at 25 mM nitrate medium with salt (Figure 3C). Next, the content of proline, which is well-known to increase in response to water stress (Greenway and Munns, 1980), was found to be higher in the *pap1-D/fls1ko* and *fls1ko* plants than in Col-0 under high nitrate and high salt conditions (Figure 3D). To investigate whether salt content differs between plants, 9-day old seedlings were supplied to 10, 25, and 50 mM KNO_3_ and 175 mM NaCl for 24 h then Na^+^ and K^+^ ion were measured, respectively (Figure 3E). The content of Na^+^ was slightly higher than that of Col-0 in 10 and 50 mM nitrate medium with salt, but no significant difference was not found in other conditions (Figure 3E). This suggests that the increased levels in anthocyanin and proline have contributed to the increase in salt stress tolerance in the *pap1-D/fls1ko* and *fls1ko* plants to some extent.

NO_3_^–^ signaling pathways are known to be closely related to sucrose; moreover carbohydrates increase anthocyanin accumulation by activating its biosynthesis genes (Meng et al., 2018). and high sucrose contents in plants cause reduction in photosynthetic efficiency (Eckstein et al., 2012). Because the anthocyanin contents were significantly higher in the *pap1-D/fls1ko* and *fls1ko* plants, we were curious about the sucrose contents in these plants. Thus, the sucrose contents were measured after 9-day-old seedlings were treated with 10, 25, and 50 mM KNO_3_ and 175 mM NaCl for 24 h (Supplementary Figure 4). Decreased sucrose contents were detected following increased NO_3_^–^ concentrations under salt stress. The *pap1-D/fls1ko* plants had lower sucrose contents than the Col-0 and *fls1ko* plants. The *fls1ko* plants showed decreased sucrose contents than the Col-0 plants but slightly higher than the *pap1-D/fls1ko* plants. In contrast, the *ttg1ko* plants showed higher sucrose contents than the Col-0 plants under 25 mM NO_3_^–^ with salt stress. These results indicate that high sucrose contents in the *ttg1ko* plants may partly contribute to better growth performance in response to high NO_3_^–^ concentrations under salt stress because high sucrose contents in plants can confer enhanced stress tolerance (Pommerrenig et al., 2018).

### NO_3_^–^ Contents and Transporter Gene Transcript Levels Were Altered in Response to High Nitrate Concentrations Under Salt Stress

**FIGURE 4:**
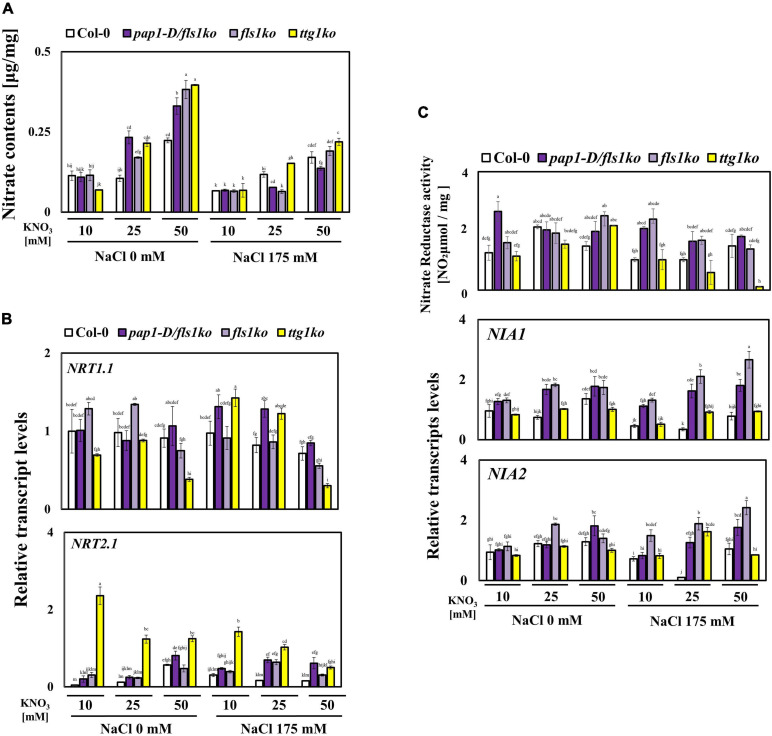
Altered NO_3_^–^ metabolism after treatment with high NO_3_^–^ concentrations under salt stress conditions. **(A)** The NO_3_^–^ contents in 9-day-old Col-0, *pap1-D/fls1ko*, *fls1ko*, and *ttg1ko* plants were measured after treatment with 10, 25, and 50 mM KNO_3_ and 175 mM NaCl for 24 h. The NO_3_^–^ content of each seedling is shown per seedling fresh weight (mg). **(B)** Nine-day-old Col-0, *pap1-D/fls1ko*, *fls1ko*, and *ttg1ko* plants were treated with 10, 25, and 50 mM KNO_3_ and 175 mM NaCl for 6 h, and the RNA was extracted. The relative transcript levels of *NRT1.1* and *NRT2.1* (nitrate transport encoding genes) were confirmed by using each cDNA sample. *Actin2* was used as an internal control. **(C)** Nine-day-old Col-0, *pap1-D/fls1ko*, *fls1ko*, and *ttg1ko* plants were treated with 10, 25, and 50 mM KNO_3_ and 175 mM NaCl for 24 h and the nitrate reductase (NR) activity was confirmed. The NR activity of each seedling is shown per seedling fresh weight (mg). For the detection of relative transcript levels of *NIA1* and *NIA2* (nitrate reductase related encoding genes), 9-day-old Col-0, *pap1-D/fls1ko*, *fls1ko*, and *ttg1ko* plants were supplied with 10, 25, and 50 mM KNO_3_ and 175 mM NaCl for 6 h and the RNA was extracted. The relative transcript levels of *NIA1* and *NIA2* were measured by using each cDNA samples and *Actin2* as internal control. Three independent experiments were conducted, and the data were subjected to a factorial ANOVA, followed by Tukey’s test (*P* < 0.05). The letters above the columns indicate significant differences. Bars represent the standard errors.

Next, we analyzed whether the NO_3_^–^ contents differed in these plants. The 9-day-old seedlings were sampled to measure the NO_3_^–^ contents after the seedlings were exposed to 10, 25, and 50 mM of NO_3_^–^ under salt stress for 24 h. As shown in Figure 4A, the NO_3_^–^ contents increased in response to high NO_3_^–^ concentrations under salt stress; however, *pap1-D/fls1ko* and *fls1ko* plants showed lower NO_3_^–^ contents than the Col-0 plants especially under 50 mM NO_3_^–^ with salt stress. Conversely, the *ttg1ko* plants showed higher NO_3_^–^ levels than the Col-0, *pap1-D/fls1ko*, and *fls1ko* plants (Figure 4A). To determine the reason for the altered NO_3_^–^ contents, the transcript levels of several NO_3_^–^ transporter genes were examined after treatment of 9-day-old seedlings with NO_3_^–^ and salt (Figure 4B). The *NRT1.1*, a widely known NO_3_^–^ transporter, showed decreased transcript levels under high NO_3_^–^ and salt conditions in Col-0 plants. In *pap1-D/fls1ko* plants, *NRT1.1* transcript levels were highly induced but showed a decreasing trend due to the high NO_3_^–^ concentration. The *fls1ko* plants showed a similar transcript level to Col-0 plants; however, the *ttg1ko* plants showed highly induced transcript levels that were similar to *pap1-D/fls1ko* plants only under 10 and 25 mM KNO_3_ with salt stress. The gene *NRT2.1*, a low-affinity NO_3_^–^ transporter, showed decreased transcript levels in Col-0 plants under high NO_3_^–^ and salt conditions; however, *pap1-D/fls1ko*, *fls1ko*, and *ttg1ko* plants showed significantly increased transcript levels (Figure 4B).

### Optimal Anthocyanin Levels Increased the Nitrogen Use Efficiency of *pap1-D/fls1ko* and *fls1ko* Plants

Since NO_3_^–^ contents were reduced in the *pap1-D/fls1ko* and *fls1ko* plants, we speculated that these plants harbored more nitrate reductase (NR) activity under high NO_3_^–^ and salt conditions. To investigate the NR activity in response to salt and various NO_3_^–^ concentrations, we conducted the NR activity assay after treating 9-day-old seedlings with 10, 25, and 50 mM NO_3_^–^ under salt stress for 24 h. As shown in Figure 4C, while the NR activities were increased in the Col-0 plants in response to high NO_3_^–^ concentrations with salt stress, the *pap1-D/fls1ko* and *fls1ko* plants showed slightly higher NR activities than Col-0 plants under all the conditions tested. However, the *ttg1ko* plants (which had much higher sucrose contents) showed extremely reduced NR activity levels compared with the Col-0 plants under high NO_3_^–^ contents with salt stress (Figure 4C). To determine if the transcript levels of nitrate reductase related genes, *NIA1* and *NIA2*, were altered, 9-day-old seedlings were separately treated with 10, 25, and 50 mM nitrate in combination with 175 mM NaCl for 24 h. The major nitrate reductase *NIA1* (*Nitrate reductase 1*) and *NIA2* (*Nitrate reductase 2*) transcript levels were measured using qRT-PCR. In Col-0 plants, *NIA1* transcript level marginally increased in response to high nitrate content under salt stress conditions (Figure 4C). Consistent with the NR activity assay results, both *pap1-D/fls1ko* and *fls1ko* plants showed highly increased *NIA1* transcript level following the exposure to high nitrate contents. The *ttg1ko* plants that had reduced NR activity, showed similar transcript level under normal nitrate concentration (10 mM), but slightly increased under 25 and 50 mM nitrate concentrations with salt stress. The *NIA2* transcript level was also increased in response to high nitrate content with salt stress in Col-0 plants and had similar levels of *NIA1* in *pap1-D/fls1ko*, *fls1ko*, and *ttg1ko* plants (Figure 4C).

**FIGURE 5:**
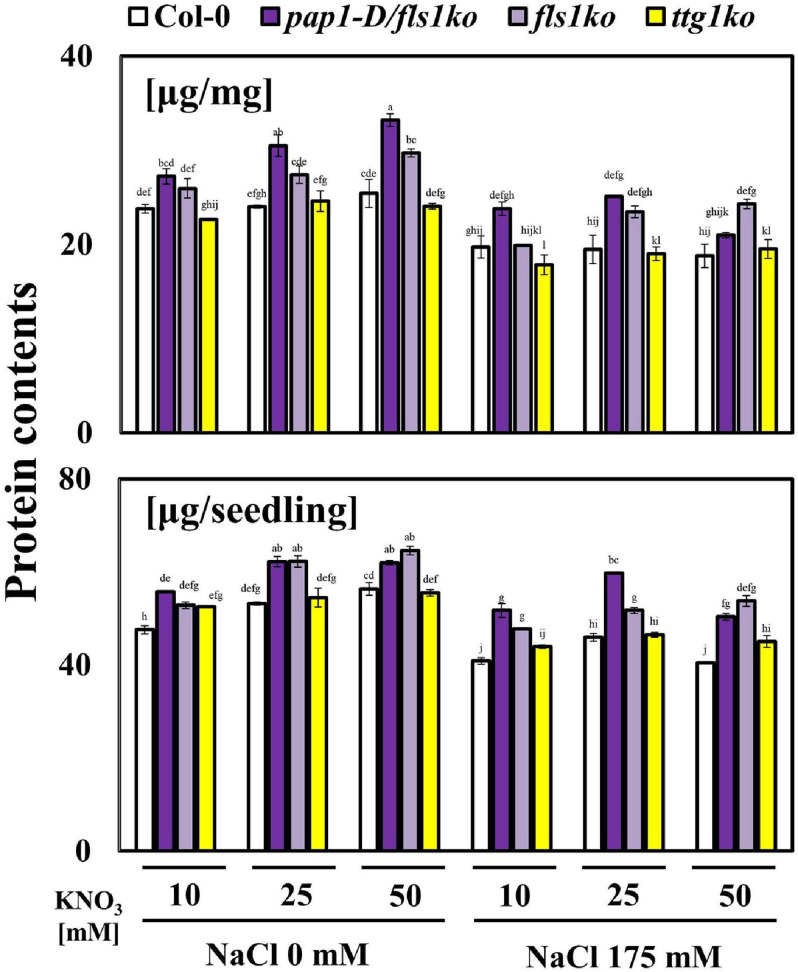
Enhanced protein contents of plants in response to high NO_3_^–^ and salt conditions. Nine-day-old seedlings were transferred to various NO_3_^–^ concentrations (KNO_3_: 10, 25, and 50 mM) and 175 mM NaCl-treated medium after growth in normal 10 mM NO_3_^–^ treated media. The protein contents were measured and are shown per seedling fresh weight (mg). Three independent experiments were conducted, and the data were subjected to a factorial ANOVA, followed by Tukey’s test (*P* < 0.05). The letters above the columns indicate significant differences. Bars represent the standard errors.

Finally, we evaluated the total protein contents in these plants to determine whether the nitrogen use efficiency was increased in *pap1-D/fls1ko* and *fls1ko* plants. As demonstrated in Figure 5, the protein contents significantly increased in *pap1-D/fls1ko* and *fls1ko* plants compared with Col-0 and *ttg1ko* plants.

## Discussion

One of the many responses observed in salt-stressed plants is a decline in general metabolic activities. We have been interested in the relationship between NO_3_^–^ metabolism and salt stress because the activity of NR enzymes is noticeably reduced when plants are exposed to salt stress (Gao et al., 2013). Since many farmers use much nitrogen fertilizer, we wanted to investigate whether high nitrogen concentrations help plants tolerate salt stress. Moreover, we aimed to investigate whether the mutant plants that over-produce anthocyanins (which help with the oxidative stress associated with salt stress) were better than Col-0 in terms of tolerance to high-salt stress in the presence of high NO_3_^–^.

To investigate the effect of high NO_3_^–^ concentrations on salt stress tolerance in plants, we fixed the salt concentration to 175 mM NaCl and provided three different concentrations of NO_3_^–^: 10, 25, or 50 mM. Under these conditions, the *fls1ko* plants showed the best growth performance among the four plant categories, although the primary root length results were not significantly different (Figures 1A,B). Without salt stress, we found that the growth performance of Col-0 plant was reduced as nitrate concentration increased, which was also observed in other plants (Saiz-Fernández et al., 2015). The authors proposed that high NO_3_^–^ inhibits maize growth by causing hormonal alterations that modify plant growth from cell to whole plant. To determine whether the anthocyanin content was higher in *pap1-D/fls1ko* plants than in *fls1ko* plants, we examined the anthocyanin content and observed that *pap1-D/fls1ko* plants accumulated more than twice as much anthocyanin than *fls1ko* plants in all conditions (Figure 2A). Since the *pap1-D/fls1ko* plants can accumulate much higher anthocyanin levels than Col-0, *fls1ko* and *ttg1ko* plants, we expected that the *pap1-D/fls1ko* plants would exhibit the best tolerance toward salt stress in combination with high NO_3_^–^ concentrations in the growth medium. However, the *fls1ko* plants that accumulated less anthocyanin than *pap1-D/fls1ko* plants showed high tolerance to salt stress with enhanced chlorophyll contents (Figure 1C). This result demonstrates that a dramatic increase in the anthocyanin contents in plants does not lead to enhanced tolerance to salt stress in *Arabidopsis*. This raises the question of why salt stress tolerance is not proportional to the anthocyanin level, although there is a large amount of anthocyanin in *pap1-D/fls1ko* plants. A plausible explanation could be found in the metabolic energy problem. In order for plants to synthesize anthocyanin, a secondary metabolite, various enzymes and transcriptional regulators are required, and excess energy is used for these reactions. Therefore, the reduced stress tolerance in *pap1-D/fls1ko* plants may be related to the consumption of excessive energy for anthocyanin synthesis and the reduction of energy sources for the various reactions required for their tolerance to salt stress. The *LBD* genes (*LBD37*, *LBD38*, and *LBD39*) are negative regulators of anthocyanin biosynthesis in *Arabidopsis*. When these genes were overexpressed in NO_3_^–^ deficient conditions, the transcript levels of *PAP1* and *PAP2*, which are key transcriptional factors for anthocyanin biosynthesis, were suppressed, leading to reduction in flavonoid levels. In contrast, *lbd37*, *lbd38*, or *lbd39* mutants accumulated anthocyanin when grown in NO_3_^–^ sufficient conditions. We observed that the transcript levels of *LBD37* and *LBD39* genes increased with an increase in NO_3_^–^ concentration, which was also seen in *pap1-D/fls1ko*, *fls1ko* and Col-0 plants (Supplementary Figure 3). Moreover, it has been reported that nitrogen controls a coordinated regulation of both positive regulators (MYB proteins) and negative regulators (LBD proteins) in the flavonoid biosynthesis pathway in plants (Soubeyrand et al., 2014). This indicates that the reduction in anthocyanin accumulation in Col-0 plants in response to high NO_3_^–^ contents with salt may not be caused by the induction of *LBD37* and *LBD39* genes. It appears that PAP1 plays a major role in the reduction of anthocyanin in Col-0 with its transcript level decreased under high NO_3_^–^ concentrations with salt stress (Figure 2B).

We subsequently examined various stress index responses to understand the reason underlying the improved growth performance under salt stress of the *pap1-D/fls1ko* and *fls1ko* plants in higher nitrate conditions. The transcript levels of *COR15b*, *DREB2A*, *KIN2*, and *RD22* which are stress marker genes were not significantly different between the tested plants (Figure 3A). In addition, DAB staining assay showed slightly weaker in the *pap1-D/fls1ko* and *fls1ko* plants in 10 mM nitrate medium than Col-0, but there was no significant difference under other conditions (Figure 3B). Since we did not measure all the various types of ROS contents, we cannot conclude that the total ROS contents of the *pap1-D/fls1ko* and *fls1ko* plants is not much different from those of Col-0. In contrast, the content of proline was found to be relatively higher in the *pap1-D/fls1ko* and *fls1ko* plants than in the Col-0 at high nitrate and high salt conditions (Figure 3D), which is probably because of the increase in the activity of nitrate reductase in these plants (Figure 4C). Therefore, it can be assumed that the higher salt stress tolerance is higher in in the *pap1-D/fls1ko* and *fls1ko* plants than in Col-0 under high nitrate and salt stress conditions is because of the higher proline contents of these plants more than Col-0. We adopted *ttg1ko* as a control plant that lacks anthocyanins to investigate the role of anthocyanin under high NO_3_^–^ and high salt conditions. At first, we expected that *ttg1ko* plants would exhibit significantly lower stress tolerance than Col-0 plants in all tested conditions because anthocyanins play important roles in plant stress tolerance. However, under 25 mM of NO_3_^–^ with salt, *ttg1ko* plants exhibited more tolerance to salt stress than Col-0 plants (Figure 1A). The high salt stress tolerance of *ttg1ko* plants could be partly attributed to the higher sucrose content (Supplementary Figure 4). Plants have established a complex regulatory mechanism, which coordinates nitrogen with carbon metabolism. The metabolites serving as substrates and products are important in controlling and facilitating the coordination of carbon metabolism and nitrogen assimilation (Nunes Nesi et al., 2010). This is necessary to prevent the excessive use of carbohydrates and avoid the accumulation of toxic products (NO_2_ and NH_4_^+^), leading to strict regulation of the various steps of nitrogen assimilation. Sucrose can control the NR activity by modulating the phosphorylation level of NR (Iglesias-Bartolomé et al., 2004). Sucrose inhibits SNRK1 (SNF1 related protein kinase), which is proposed to phosphorylate and inhibit NR (Lillo, 2008). When the concentration of nitrogen in plants is low, the anthocyanin content increases. When the concentration of nitrogen is lowered, the relative proportion of carbohydrates increases (Scheible et al., 2004). Carbohydrates, particularly sucrose, induce anthocyanin accumulation by modulating the MBW complex genes in various plant species (Payyavula et al., 2013; Xi et al., 2018). Higher carbohydrate contents in plants can lead to problems such as lower photosynthesis efficiency. To prevent this, plants increase the synthesis of anthocyanins to lower the carbohydrate contents. In contrast, when nitrogen is abundant, carbohydrates are needed for the production of amino acids and other macromolecules; hence, anthocyanin biosynthesis is suppressed.

We observed that when the growth medium contained high NO_3_^–^ concentrations, the NO_3_^–^ content increased inside the plant cells (Figure 4A). However, NO_3_^–^ contents of both *pap1-D/fls1ko* and *fls1ko* plants under 25 and 50 mM NO_3_^–^ concentrations with salt stress conditions were lower than those of Col-0 plants, and the NO_3_^–^ contents in the *ttg1ko* plants were the highest under 10 and 50 mM NO_3_^–^ concentrations with salt stress conditions (Figure 4A). In these plants, the decrease in the transcript levels of the NO_3_^–^ transporter genes does not seem to have caused reduction in the NO_3_^–^ content in the cells because when compared with these mutant and Col-0 plants, the transcript levels of NO_3_^–^ transporter genes such as the *NRT1.1* and *NRT2.1* were similar or greater in the *pap1-D/fls1ko, fls1ko* and *ttg1ko* plants (Figure 4B). Although the transcript levels of the NO_3_^–^ transporter genes were not significantly different, the NO_3_^–^ contents were low in the *pap1-D/fls1ko* and *fls1ko* plants. Thus, we explored whether there was any difference in the activity of the NR enzymes among these plants. This increase in the NR activity might lead to an increase in protein synthesis in the *pap1-D/fls1ko* and *fls1ko* plants (Figure 4C). This is presumed to have contributed to the increase in proline content (Figure 3D). Our results show that the increased accumulation of anthocyanins in plants can partly contribute to improving the nitrogen use efficiency of stressed plants. Anthocyanins function as excellent antioxidants; therefore, we predicted that the content of DAB staining would be weaker in the *pap1-D/fls1ko* and *fls1ko* plants than in Col-0, but the results did not support this (Figure 3B). However, since anthocyanin is well-known for their antioxidant activity, we think that nitrate metabolism operate more efficiently and ultimately leading to better growth performance in in the *pap1-D/fls1ko* and *fls1ko* plants than Col-0 under stressful environments owing to reduced oxidative stress.

Taken together, our results led us to conclude that salt stress tolerance of plants was weakened by high NO_3_^–^ concentrations. Although our study was performed on the model plant *Arabidopsis* and the response of other crops may be different, our findings are still a reminder that we need to minimize the use of nitrogen fertilizers in agricultural activities. Our *FLS1*-related study suggests a plant biotechnological approach to develop highly stress-resistant crops in the future. In other words, the unconditional increase in anthocyanin does not create the most effect on improving the salt stress tolerance of plants. We observed that reduction in salt stress tolerance by high NO_3_^–^ application level can be improved to some extent by increasing the anthocyanin contents (Figure 2A). Therefore, anthocyanin biosynthetic genes or other genes need to be carefully engineered to obtain plants that show enhanced tolerance against salt stress. Recently, many studies on the improvement of crop characteristics have been conducted due to the development of CRISPR technology. However, unlike conventional overexpression studies, it is now necessary to delete genes to improve specific traits of crops. The *FLS1* gene studied here could be an ideal target gene that can be modified using CRISPR technology.

## Data Availability Statement

The datasets presented in this study can be found in online repositories. The names of the repository/repositories and accession number(s) can be found in the article/Supplementary Material.

## Author Contributions

YL: anthocyanin measurement, BCA assay, determination of sucrose content, nitrate reductase assay, nitrate-salt stress phenotype assay, and qRT-PCR. WL: qRT-PCR, DAB staining, sodium and potassium measurement, proline assay, and manuscript writing. QL: chlorophyll assay and nitrate content assay. S-WH: qRT-PCR. HL: experiment design and manuscript writing. All authors contributed to the article and approved the submitted version.

## Conflict of Interest

The authors declare that the research was conducted in the absence of any commercial or financial relationships that could be construed as a potential conflict of interest.

## Footnotes

1 http://www.arabidopsis.org/

2 www.arabidopsis.org

## Supplementary Material

Supplementary Figure 1 Growth phenotype of Col-0 in response to various concentrations of potassium chloride (KCl) medium. After their growth in normal 10 mM NO_3_^–^ media, 9-day-old Col-0 plants were transferred to the 5 mM NO_3_^–^ medium supplemented with 5, 15, 25, or 45 mM KCl medium as a control for the high level NO_3_^–^ treatment. The pictures were taken 7 days after the transfer.

Supplementary Figure 2 Anthocyanin contents in response to various concentrations of potassium chloride (KCl) medium with nitrate and salt stress conditions. The anthocyanin contents were measured in 4-day-old Col-0, *pap1-D/fls1ko*, *fls1ko*, and *ttg1ko* plants after treatment with 10, 25, and 50 mM KNO_3_ and 175 mM NaCl for 24 h by color-spectrometric absorbance. For each medium, KCl was added to ensure the KCl concentration was uniform.

Supplementary Figure 3 Anthocyanin biosynthesis inhibition-related genes transcript levels of plants after treatment with high concentrations of NO_3_^–^ under salt stress conditions. Nine-day-old Col-0, *pap1-D/fls1ko*, *fls1ko*, and *ttg1ko* plants were treated with 10, 25, and 50 mM KNO_3_ and 175 mM NaCl for 6 h and the RNA was extracted. The relative transcription levels of the anthocyanin biosynthesis inhibition-related genes (*LBD37*, *LBD38*, and *LBD39*) and anthocyanin biosynthesis inhibition related gene *MYBL2* were confirmed using each cDNA sample. *ACTIN2* was used as an internal control. Three independent experiments were conducted, and the data were subjected to a factorial ANOVA, followed by Tukey’s test (*P* < 0.05). The letters above the columns indicate significant differences. Bars represent the standard errors.

Supplementary Figure 4 Significantly decreased sucrose contents in *pap1-D/fls1ko* and *fls1ko* plants under high NO_3_^–^ and salt conditions. Nine-day-old Col-0, *pap1-D/fls1ko*, *fls1ko*, and *ttg1ko* plants were transferred to 10, and 25 mM KNO_3_ treated control media after growth in normal 10 mM NO_3_^–^ media. The sucrose contents were then measured from each seedling. Three independent experiments were conducted, and the data were subjected to Tukey’s test (*P* < 0.05). The letters above the columns indicate significant differences. Bars represent the standard errors.

Supplementary Table 1 List of primers designed for qRT-PCR.
