# Supplementary figures and images for: Growth Performance Can Be Increased Under High Nitrate and High Salt Stress Through Enhanced Nitrate Reductase Activity in Arabidopsis Anthocyanin Over-Producing Mutant Plants
Source: Front Plant Sci. 2021 Jul 1;12:644455. doi: 10.3389/fpls.2021.644455 (PMC8280297; doi:10.3389/fpls.2021.644455)

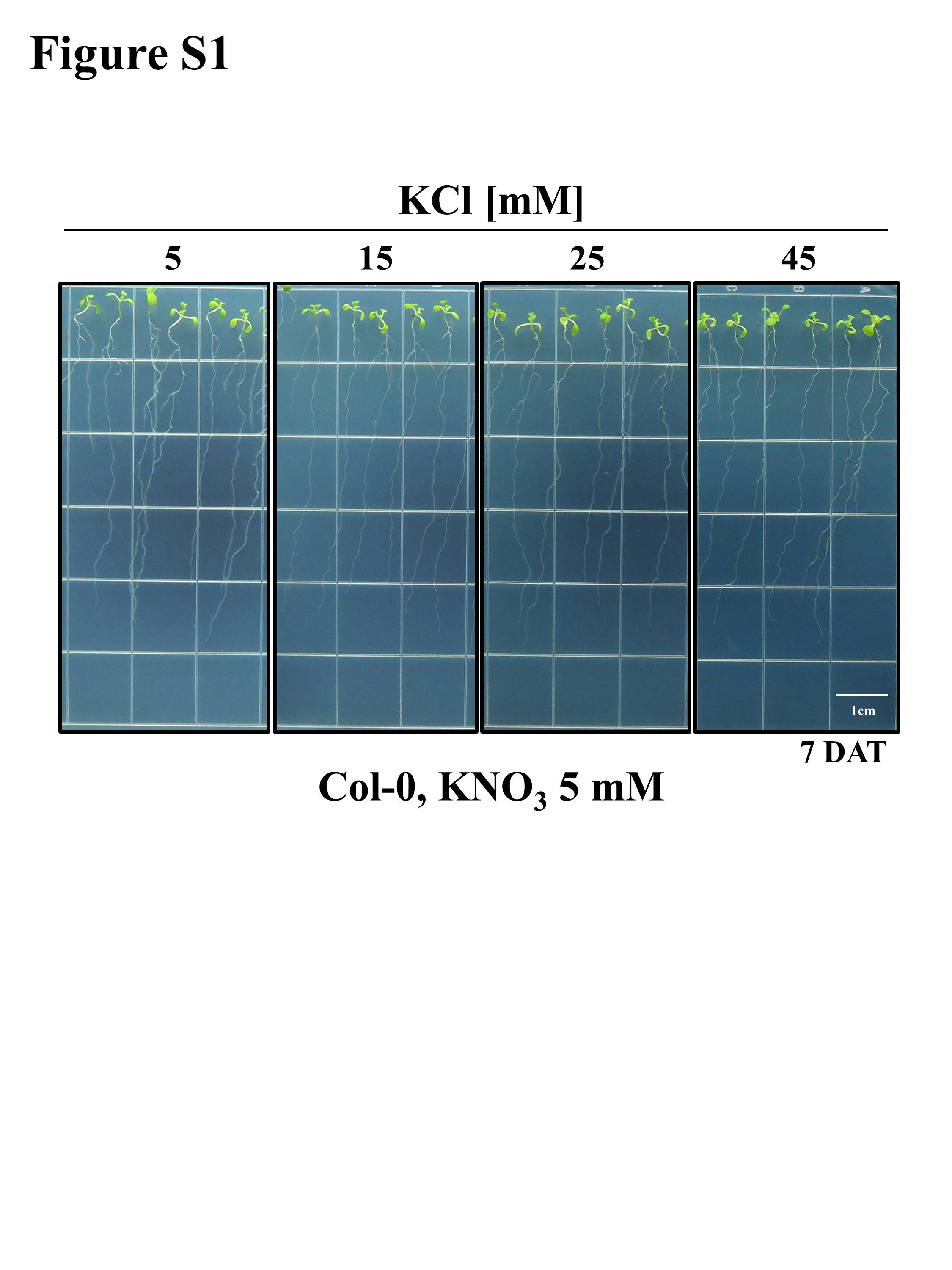

Supplement: Supplementary Figure 1 — Growth phenotype of Col-0 in response to various concentrations of potassium chloride (KCl) medium. After their growth in normal 10 mM NO3– media, 9-day-old Col-0 plants were transferred to the 5 mM NO3– medium supplemented with 5, 15, 25, or 45 mM KCl medium as a control for the high level NO3– treatment. The pictures were taken 7 days after the transfer. [file Image_1.JPEG]

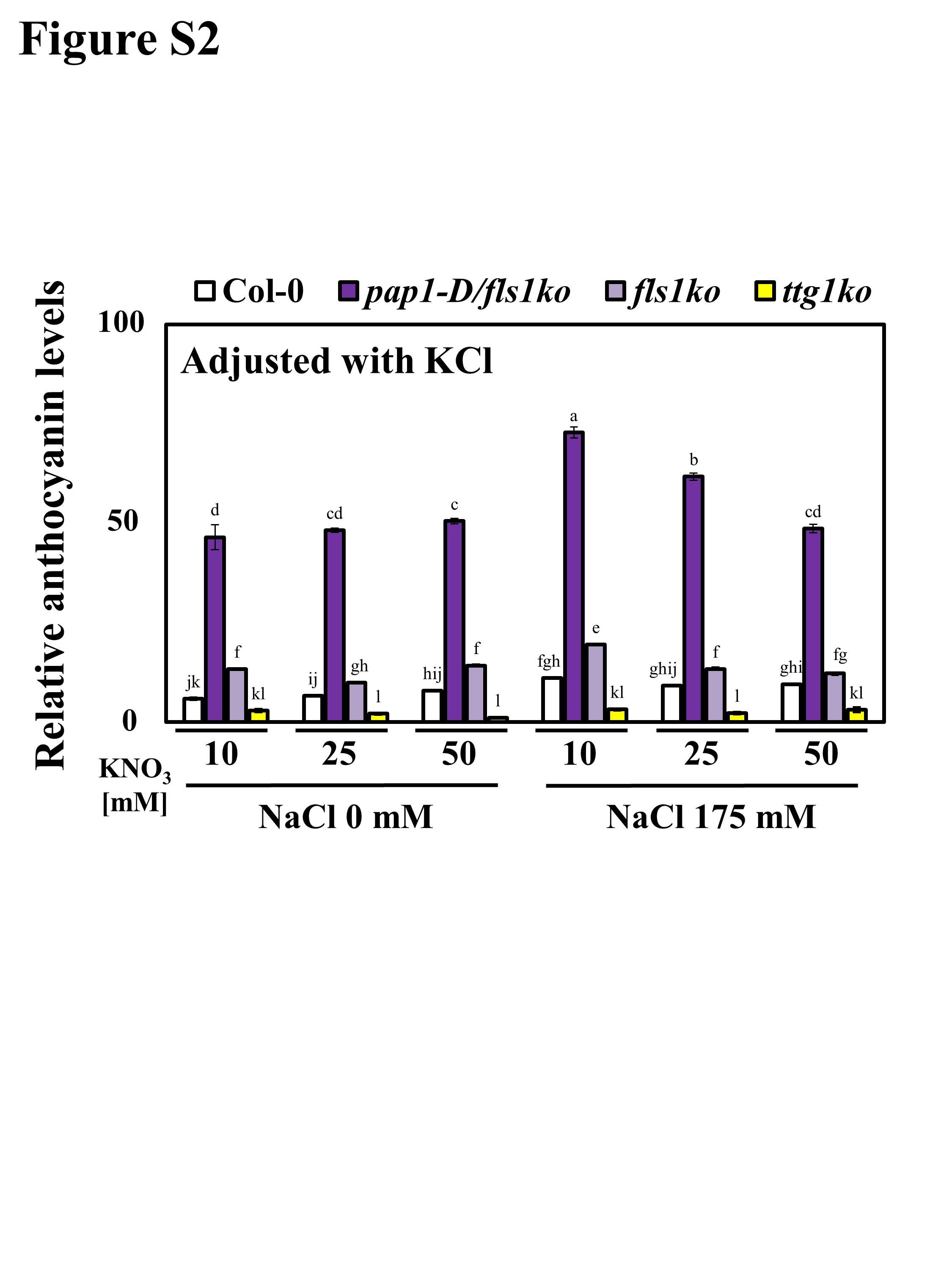

Supplement: Supplementary Figure 2 — Anthocyanin contents in response to various concentrations of potassium chloride (KCl) medium with nitrate and salt stress conditions. The anthocyanin contents were measured in 4-day-old Col-0, pap1-D/fls1ko, fls1ko, and ttg1ko plants after treatment with 10, 25, and 50 mM KNO3 and 175 mM NaCl for 24 h by color-spectrometric absorbance. For each medium, KCl was added to ensure the KCl concentration was uniform. [file Image_2.JPEG]

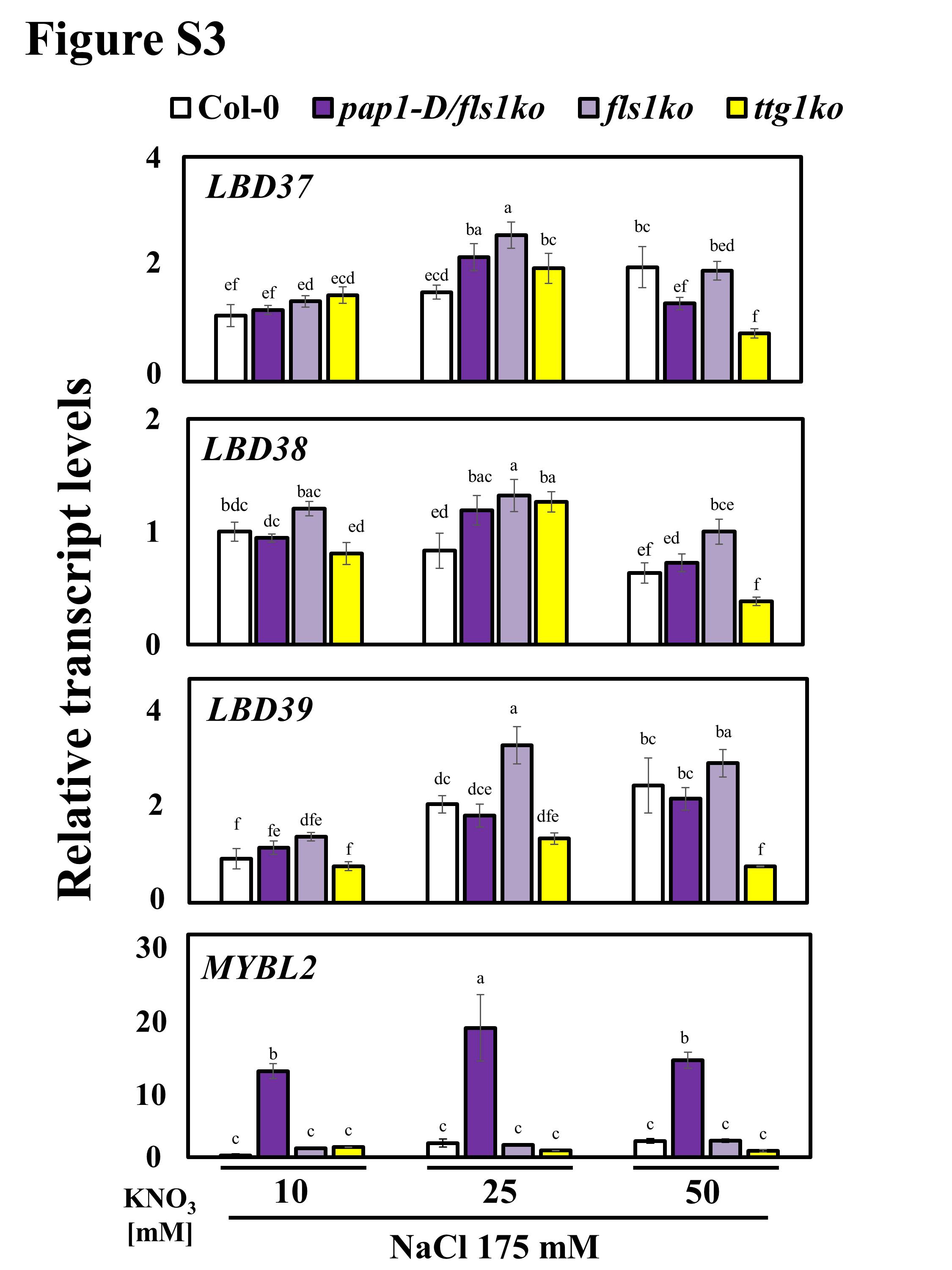

Supplement: Supplementary Figure 3 — Anthocyanin biosynthesis inhibition-related genes transcript levels of plants after treatment with high concentrations of NO3– under salt stress conditions. Nine-day-old Col-0, pap1-D/fls1ko, fls1ko, and ttg1ko plants were treated with 10, 25, and 50 mM KNO3 and 175 mM NaCl for 6 h and the RNA was extracted. The relative transcription levels of the anthocyanin biosynthesis inhibition-related genes (LBD37, LBD38, and LBD39) and anthocyanin biosynthesis inhibition related gene MYBL2 were confirmed using each cDNA sample. ACTIN2 was used as an internal control. Three independent experiments were conducted, and the data were subjected to a factorial ANOVA, followed by Tukey’s test (P < 0.05). The letters above the columns indicate significant differences. Bars represent the standard errors. [file Image_3.JPEG]

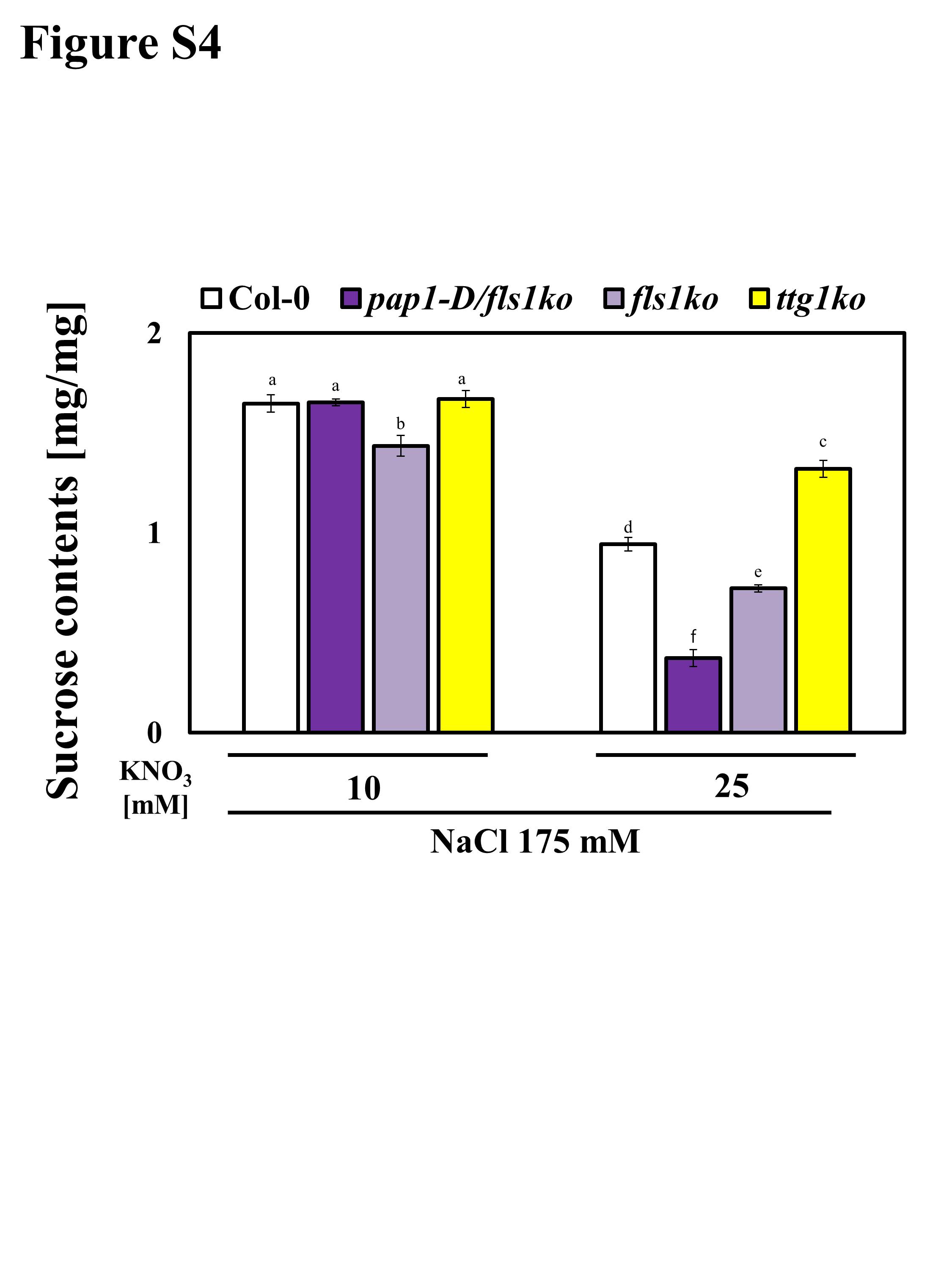

Supplement: Supplementary Figure 4 — Significantly decreased sucrose contents in pap1-D/fls1ko and fls1ko plants under high NO3– and salt conditions. Nine-day-old Col-0, pap1-D/fls1ko, fls1ko, and ttg1ko plants were transferred to 10, and 25 mM KNO3 treated control media after growth in normal 10 mM NO3– media. The sucrose contents were then measured from each seedling. Three independent experiments were conducted, and the data were subjected to Tukey’s test (P < 0.05). The letters above the columns indicate significant differences. Bars represent the standard errors. [file Image_4.JPEG]
